# Self-reported competence, attitude and approach of physicians towards patients with dementia in ambulatory care: Results of a postal survey

**DOI:** 10.1186/1472-6963-8-54

**Published:** 2008-03-06

**Authors:** Hanna Kaduszkiewicz, Birgitt Wiese, Hendrik van den Bussche

**Affiliations:** 1 Department of Primary Medical Care, University Medical Centre Hamburg-Eppendorf, Hamburg, Germany; 2 Centre for Biometry, Medical Informatics and Medical Technology, Hannover Medical School, Hannover, Germany

## Abstract

**Background:**

Caring for patients with dementia is a demanding task. Little is known as to whether physicians feel competent enough to perform this task or whether a lack of self-perceived competence influences attitudes and professional approach. Even less is known with respect to potential differences between general practitioners (GPs) and specialists. The purpose of this study was to investigate the interrelationship between the self-perceived competence, attitude and professional approach of physicians in ambulatory care in Germany. A further aim was to compare GPs and specialists with regard to differences in these areas.

**Methods:**

A standardised postal survey was sent to 389 GPs and 239 neurologists and psychiatrists in six metropolitan areas in Germany. The 49-item questionnaire consisted of attitudinal statements to be rated on a Likert-type scale. Return rates were 54 percent for GPs and 40 percent for specialists. Statistical methods used to analyze data included correlation analysis, cluster analysis and ordinal regression analysis.

**Results:**

No differences were found between GPs and specialists with regard to their general attitude towards caring for patients with dementia. Approximately 15 percent of both disciplines showed a clearly negative attitude. Self-reported competence was strongly associated with general attitude. In particular among GPs, and less so among specialists, a strong positive association was found between self-reported competence, general attitude and professional approach (e.g. early detection, active case finding and cooperation with caregivers). Differences between GPs and specialists were smaller than expected and appear to predominantly reflect task differences within the German health care system.

**Conclusion:**

Training opportunities which enable in particular GPs to enhance not only their competence but also their general attitude towards dementia care would appear to be beneficial and might carry positive consequences for patients and their caregivers.

## Background

### Introduction

In most industrialized countries, GPs are responsible for providing care for the majority of patients suffering from dementia, whether in the community or in nursing homes. But do they feel competent enough to perform this task? Furthermore, does a lack of self-perceived competence influence professional attitude or approach in caring for patients with dementia? Several studies from the United Kingdom reveal that general practitioners (GPs) consider themselves to be insufficiently qualified in dealing with dementia and also see the need for improved training. In a study conducted on behalf of the Audit Commission in England and Wales, only half of the GPs surveyed felt that they had received sufficient training in diagnosing and managing dementia [1]. In a recent survey carried out in London and Scotland, one-third of the GPs expressed limited confidence in their diagnostic skills and two-thirds lacked confidence in managing behavioural problems in dementia patients [2]. These results from the UK are supported by a Swedish study in which 71 percent of GPs expressed the need for more continuing education in the field of dementia [3].

Knowledge tests have also shown dementia-related knowledge deficiencies in GPs, although results widely vary. In an earlier test conducted in Australia in 1992, three-quarters of GPs were able to cite a maximum of only two dementia symptoms [4]. Another early study conducted in Alabama revealed knowledge differences between GPs and experts in the care of Alzheimer's disease (AD) patients [5]. A study carried out at a later date with the same test showed significant differences in knowledge between older and younger physicians in the USA [6]. More recent studies have lead to more favourable test results: in the London-Scotland study cited above, GPs' knowledge test results were good [2], as was also the case in the aforementioned Swedish study [3].

Differences in self-reported competence and attitude might be due to formal qualification, e.g. according to medical speciality. In the USA, for example, Brown et al. found significantly better knowledge scores for internists than for GPs [6]. De Lepeleire et al. compared the self-reported attitudes to disclosure and the self-reported disclosure behaviour of Flemish GPs with those of a small sample of old-age psychiatrists and geriatricians in Nottingham, UK. They found comparable attitudes towards the advantages of disclosure and a somewhat lower rate of self-reported disclosure practice in the GP sample [7]. Wolff et al. also report a more positive attitude towards dementia care in a small sample of old age psychiatrists as compared to GPs [8]. As far as the authors are aware, no further studies have compared GPs and specialists with respect to caring for dementia patients.

Besides knowledge based on formal training, professional experience – as represented by the number of years in ambulatory care – might play a role in the physician's approach to dementia care, as demonstrated by Brown et al. [6]. In addition, physicians' gender might also be of importance. A number of studies suggest that female physicians possess superior communication skills [9], attach more importance to psychosocial aspects of care [10,11] and better integrate patients in the decision-making process [12].

Is professional approach to dementia care determined by the subjective competence level, knowledge, professional experience, gender or attitude of the physician? Boise et al. summarized that „attitudes rather than knowledge were the key determinants of whether the physicians conducted a full assessment (for dementia)" [13]. It would seem reasonable to assume that these factors are interrelated [6,14].

### Objectives

The main aim of this study was therefore to explore the interrelation between physicians' self-estimated competence with respect to the ambulatory care of dementia patients, their general attitude towards this care and their professional approach to the disease and its management, as exemplified by case finding, diagnosis disclosure, interaction with care-giving relatives, the value of self-help services, clinical guidelines and continuing education. A further aim was to compare GPs and specialists working in ambulatory care (neurologists and psychiatrists) with respect to each of these aspects.

The study was based on the following hypotheses:

- There is a positive interrelation between the self-estimated competence of physicians and their general attitude towards caring for demented patients.

- Knowledge based on formal training, professional experience and gender influence self-estimated competence and general attitude towards caring for demented patients.

- Both the self-estimated competence of physicians and their general attitude towards dementia care are associated with their professional approach.

**Figure 1:**
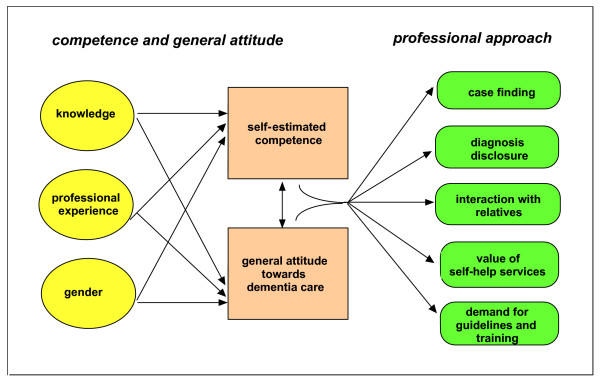
Theoretical framework of the study.

The theoretical framework of the study is depicted in Figure 1.

## Methods

A standardised 49-item self-administered questionnaire was developed on the basis of 30 semi-standardised face-to-face interviews with GPs in Hamburg and Düsseldorf. The questionnaire covered the following aspects of ambulatory care in dementia: general attitude towards dementia care, detection process, disclosure of diagnosis, therapy options, interaction with caregivers, the value of self-help services, the interest in guidelines and continuing education, and cooperation between professionals. The present paper is limited to those items on general attitude, detection process, disclosure, caregivers, self-help, guidelines and continuing education. Results on remaining aspects will be published at a later date. The questionnaire consisted of positively or negatively worded attitudinal statements to be rated on a six-point Likert-type scale (1 = minimum, 6 = maximum). In the descriptive analyses presented here, scores were partly grouped to rank the degree of consent/dissent (1 + 2 = "do not agree at all"; 3 + 4 = "partially agree"; 5 + 6 = "strongly agree"). It is assumed that participants needed some 15 minutes to complete the questionnaire.

Ten GPs piloted the questionnaire and commented on its content. The questionnaire was sent to all 129 GPs participating in the German Competence Network Dementia (CND; return rate 84 percent), 260 randomly selected GPs excluding those who participated in the interviews described above (return rate = 40 percent) and 239 randomly selected neurologists and psychiatrists working in ambulatory care (return rate = 40 percent). The study was conducted in six large cities (Hamburg, Düsseldorf, Bonn, Leipzig, Mannheim and Munich). The figures stated above represent return rates achieved after one telephone reminder. Due to the fact that a specialist in psychiatry or neurology usually spends a prolonged period of postgraduate training in the sister discipline, the boundaries between the two disciplines are blurred. In the present paper, the term "specialists" is therefore used to denote both professional groups. Since no differences were found between the GPs belonging to and not belonging to the German Competence Network Dementia with respect to self-reported competence or attitudes, results are presented as a comparison between GPs and specialists.

Statistical analysis was performed using SPSS version 14.0 and S-Plus version 7.0. For quantitative parameters (e.g. the competence index), comparisons between samples were based on the Mann-Whitney-U test (MWU). The Linear Trend Test was applied to test for trends in ordinal categories between two groups. In order to analyze relationships between quantitative variables or ordered categories, Kendall's τ test was employed. Using the Partitioning Around Medoids [15] algorithm, a cluster analysis was performed in order to identify sub-samples of physicians with similar general attitudes towards dementia care. Furthermore, an ordinal regression analysis was performed. Due to the explorative character of the analyses, no multiple test adjustments were made. However, only *p*-values ≤ 0.005 were considered significant.

The study was carried out in compliance with the Helsinki Declaration. All participants were volunteers and informed about the aims of the project. They gave informed consent by answering the questionnaire and sending it back. The study was approved by the Ethics committee of the Hamburg Medical Association (OB/8/02).

## Results

Approximately one-third of the survey participants in each sample were women. This fraction corresponds to the actual representation of female physicians in ambulatory care. The median number of working years in ambulatory care was 13 for GPs (mean 13.8 ± 7.3) and 7 for specialists (mean 9.8 ± 7.0). 38 percent of the specialists failed to reply to this question. The GPs reported simultaneously caring for 15 dementia patients on their premises and 10 in nursing homes (median values). Specialists saw an average of 40 patients exclusively on their premises (median value).

### Self-estimated competence with regard to dementia care

**Figure 2:**
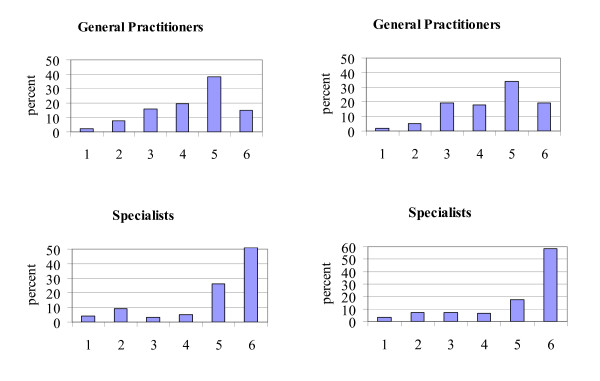
Histograms of subjective qualification regarding diagnosis (left) and therapy (right) of dementia by profession. 1 = poor, 6 = very good.

Participants were questioned regarding the extent to which they felt competent in diagnosing and treating dementia patients. Both professions reported feeling competent with regard to both diagnosis and therapy: on a six point Likert-type scale, the mean score for diagnostic competence was 4.31, and 4.38 for therapeutic competence for GPs (medians 5), and 4.95 for diagnostic competence and 5.03 for therapeutic competence for specialists (medians 6). Differences between specialists and GPs were statistically significant (*p *< 0.001, MWU). The correlation between diagnostic and therapeutic competence estimates was high in both professional groups, though especially so in the GP sample (Kendall's τ for GPs = 0.589, *p *< 0.001; for specialists = 0.493, *p *< 0.001). Distributions for both samples are shown in Figure 2. A broadly spread distribution was found among GPs and almost exclusively high scores among specialists.

An overall measure of subjective competence – hereafter referred to as competence index (CI) – was calculated as the mean score of the two competence items. The competence index was significantly higher for specialists than for GPs (median 4.5 for GPs and 5.5 for specialists; *p *< 0.001, MWU).

### General attitude towards care for patients with dementia

The general attitude of the two professional samples towards dementia care was investigated by means of four items (Items 1 – 4 in Table 1). In both professions, the distribution figures in Table 1 clearly show a general attitude that is characterized by dedication, concern and contentment with the task of caring for dementia patients. In a cluster analysis based on these four items, three types of physicians were identified, each with a distinctive general attitude:

**Table 1:** Distribution figures and associations between items in the questionnaire by profession

		**General practitioners**					**Specialists**					
			
		**Mean (SD) Median**	**association with CI**		**association with general attitude cluster**		**Mean (SD) Median**	**association with CI**		**association with general attitude cluster**		***p_3_***
						
			τ	***p_1_***	τ	***p_2_***		τ	***p_1_***	τ	***p_2_***	
1	As a physician I cannot do anything to improve the quality of life of patients with dementia	1.95 (1.20) 2	-0.321	**< 0.001**	**-**	**-**	1.83 (1.21) 1	-0.274	**0.002**	**-**	**-**	0.317
2	Caring for patients with dementia is a rewarding task for me as a physician	3.56 (1.31) 4	0.239	**< 0.001**	**-**	**-**	3.56 (1.41) 4	0.228	**0.005**	**-**	**-**	0.998
3	I feel helpless in the relationship with my demented patients	2.63 (1.30) 2	-0.270	**< 0.001**	**-**	**-**	2.59 (1.24) 2	-0.238	**0.004**	**-**	**-**	0.906
4	I would prefer to have nothing to do with the care for dementia patients	1.97 (1.24) 1	-0.342	**< 0.001**	**-**	**-**	1.74 (1.26) 1	-0.244	**0.005**	**-**	**-**	0.032
5	Early detection of dementia benefits the patient	5.00 (1.23) 5	0.248	**< 0.001**	-0.314	**< 0.001**	5.05 (1.27) 6	0.067	0.433	-0.256	**0.005**	0.546
6	Early detection of dementia has no therapeutic consequences.	1.88 (1.28) 1	-0.195	**0.001**	0.302	**< 0.001**	1.66 (1.24) 1	-0.028	0.754	0.283	**0.003**	0.069
7	I actively search for dementia in all patients over 65	3.16 (1.48) 3	0.238	**< 0.001**	-0.195	**0.001**	3.43 (1.74) 3	0.145	0.074	-0.248	**0.005**	0.201
8	In case of suspicion of cognitive problems I regularly use cognitive tests	3.80 (1.81) 4	0.367	**< 0.001**	-0.162	**0.005**	5.06 (1.58) 6	0.329	**< 0.001**	-0.267	**0.004**	< 0.001
9	I suggest to the relatives that they contact the Alzheimer Association	4.10 (1.68) 5	0.020	0.716	-0.048	0.418	4.67 (1.44) 5	0.105	0.210	-0.194	0.031	0.005
10	I suggest to the relatives to participate in a self-help group	4.47 (1.50) 5	0.179	**0.001**	-0.149	0.012	4.80 (1.28) 5	0.119	0.158	-0.205	0.024	0.079
11	I propose the relatives often to help them in organising the care, e.g. in finding a legal guardian	4.60 (1.35) 5	0.206	**< 0.001**	-0.284	**< 0.001**	4.74 (1.20) 5	0.018	0.830	-0.055	0.544	0.630
12	To my opinion, the relatives have exaggerated communication needs	2.53 (1.50) 2	-0.217	**< 0.001**	0.380	**< 0.001**	2.63 (1.45) 2	0.033	0.687	0.110	0.218	0.441
13	Usually I can help relatives with burden quite well	4.32 (1.02) 4	0.183	**0.001**	-0.326	**< 0.001**	4.26 (1.06) 4	-0.045	0.590	-0.177	0.050	0.780
14	Guidelines for the diagnosis and treatment of dementia would help me	4.50 (1.49) 5	-0.141	0.010	0.004	0.949	3.79 (1.52) 4	-0.206	0.012	0.029	0.739	< 0.001
15	I would like to participate in a training on how to deal and speak with demented patients and their relatives	4.13 (1.60) 5	-0.138	0.011	0.072	0.224	3.35 (1.69) 3	-0.092	0.256	-0.026	0.767	< 0.001

CI = competence index; SD = standard deviation, n = number of respondents, *p*_1 _= statistical significance of association between competence index and items; *p*_2_, statistical significance of association between general attitude cluster and items; *p*_3 _= statistical significance of difference between GPs and specialists; n = 211 for GPs and 96 for specialists.

- Cluster 1: physicians with an explicitly positive general attitude towards caring for patients with dementia (reported a belief in their ability to improve the patients' quality of life, no feelings of helplessness and a view of the care of dementia patients as rewarding and further denied preferring having nothing to do with the care of such patients).

- Cluster 2: physicians with a less positive and a partly negative general attitude towards caring for patients with dementia as compared to Cluster 1.

- Cluster 3: physicians with a clearly negative general attitude towards caring for patients with dementia (reported a lack of belief in their ability to improve the patients' quality of life, explicit feelings of helplessness, etc.).

**Figure 3:**
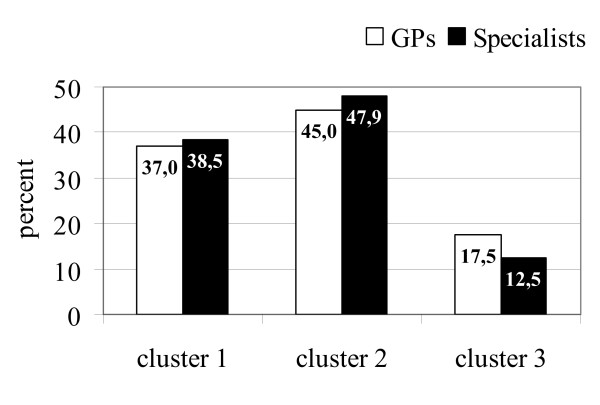
Distribution of GPs (N = 211) and specialists (N = 96) over the general attitude clusters.

Comparable distributions were found across the three clusters for both professional groups, with those physicians reporting a negative or "nihilistic" general attitude clearly representing a minority (see Figure 3). While GPs were more greatly represented in the negative sample (Cluster 3), the difference between GPs and specialists was not significant (*p *= 0.434, Linear Trend Test).

### Interrelationship between competence and general attitude

An association was found between the competence index and the general attitude items (1–4) for both groups, with a weaker correlation for the specialist-group (see Table 1). This positive association is also clearly evident when comparing competence figures and attitude clusters (Table 2): both means and medians of the competence index significantly decrease from Cluster 1 through to Cluster 3 (*p *< 0.001 for both professional groups, Kendall's τ).

**Table 2:** Association of competence index (CI) and general attitude cluster by speciality

**General attitude cluster**	**CI figures for general practitioners**			**CI figures for specialists**		
	
	**median**	**mean**	**SD**	**median**	**mean**	**SD**
1: positive	5.0	4.8	1.06	6.0	5.5	1.03
2: intermediate	4.5	4.4	0.97	5.0	4.8	1.18
3: negative	3.5	3.3	1.03	5.0	4.3	1.78

### Relationship between competence, general attitude and professional approach

The general question under investigation was whether a high estimate of competence and a positive general attitude towards dementia care are reflected in the professional approach of physicians.

### Early detection and case finding behaviour

Highly positive attitudes towards the early detection of dementia and the presumed therapeutic consequences were found in both samples (Items 5 and 6 in Table 1). These attitudes were strongly associated with the competence index in the GP sample and with general attitude towards dementia care in both samples.

However, active case finding in daily practice proved to be the exception rather than the rule (Table 1, Item 7): Only 20 percent of the GPs and 34 percent of the specialists strongly confirmed "actively searching for dementia in all patients over the age of 65" (*p *= 0.201, MWU). Active case finding was strongly associated with the competence index in the GP-sample and with general attitude towards dementia care in both samples.

Significant differences between the two professional groups were also found with respect to the use of cognitive tests in the case of suspected dementia (Table 1, Item 8): 79 percent of specialists and only 43 percent of GPs strongly agreed to this item (*p *< 0.001, MWU). The use of tests was strongly associated with the competence index and the general attitude towards dementia care in both professional groups.

In summary, GPs with a higher level of self-perceived competence and a positive general attitude towards dementia care were more in favour of early detection, assumed therapeutic possibilities, more actively looked for dementia in patients over the age of 65 and more frequently used cognitive tests than those with a lower level of self-reported competence and a less positive general attitude (*p *≤ 0.005 for all items, Kendall's τ). For specialists, similar associations were found for general attitude towards dementia but not for the competence index, with the exception of the application of cognitive tests.

### Diagnosis disclosure behaviour

The two professional groups were equally and greatly in favour of an early diagnosis disclosure: 77 percent of the specialists and 70 percent of the GPs strongly confirmed that "patients with dementia should be informed at an early stage in order to enable them to plan their lives". An examination of the relationship between the competence index and diagnosis disclosure behaviour revealed a varied picture. GPs who reported feeling highly competent were more in favour of disclosure (Items 19, 20 and 24 in Table 3), though not for disclosure using the terms "Alzheimer" or "dementia" (Items 21 and 22 in Table 3). For specialists, an almost inverse relationship was found: there was no association between self-estimated competence and favouring early disclosure, although a statistically significant association was observed with regard to the use of specific terms.

**Table 3:** Association of diagnosis disclosure items with competence index and general attitude towards dementia care by specialities

		**General practitioners**				**Specialists**			
		**Association with CI**		**Association with general attitude cluster**		**Association with CI**		**Association with general attitude cluster**	

		τ	***p_1_***	τ	***p_2_***	τ	***p_1_***	τ	***p_2_***

16	Disclosing diagnosis and prognosis does more harm than good to the patient	-0.770	0.159	0.106	0.075	-0.099	0.238	0.128	0.157
17	Most patients are grateful when I address their cognitive decline	0.137	0.011	-0.219	**< 0.001**	0.123	0.140	-0.232	0.010
18	Patients react with shame when their cognitive deficits are addressed	-0.096	0.074	0.130	0.028	-0.102	0.220	0.196	0.029
19	I only disclose when the patient demands it	-0.232	**< 0.001**	0.135	0.024	-0.019	0.818	0.035	0.701
20	Patients with dementia should be informed because of their possibility to plan their lives	0.228	**< 0.001**	-0.149	0.015	0.180	0.038	-0.212	0.024
21	When communicating the diagnosis to the patient I never use the term dementia	-0.41	0.450	0.069	0.243	-0.228	**0.005**	0.198	0.024
22	When communicating the diagnosis to the patient I never use the term Alzheimer	-0.017	0.753	0.041	0.479	-0.245	**0.003**	0.210	0.017
23	I inform the relatives more than the patient on the course of the disease	0.113	0.041	-0.126	0.038	-0.040	0.636	0.009	0.919
24	In relation to the relatives I avoid the diagnosis and I prefer to use terms like „senility" or „perfusion problems"	-0.157	**0.005**	0.128	0.037	-0.188	0.028	0.183	0.046

CI = competence index; *p*_1 _= statistical significance of association between competence index and items; *p*_2, _= statistical significance of association between general attitude cluster and items

No association was found between general attitude towards dementia care and diagnosis disclosure in either sample, with the exception of a positive correlation at the 0.005%-level in the GP-sample for the view that most patients are grateful when their cognitive decline is addressed (Table 3, Item 17). In summary, the attitude towards diagnosis disclosure and the handling of disclosure were only weakly associated with both self-estimated professional competence and general attitude towards dementia care.

### Interaction with relatives

Items 11 – 13 in Table 1 show a similarly positive attitude towards the support of relatives (*p *= n.s. for all items; MWU) in both professions. A supportive and communicative attitude was related to self-reported competence and general attitude towards dementia care in the GP sample (*p *≤ 0.001 for all items, Kendall's τ), whereas no such associations were found for the specialists.

### The role of self-help and voluntary services

The large majority of physicians – 60 percent of the GPs and 70 percent of the specialists – also reported "suggesting participation in a self-help group to the relatives" (*p *= 0.08; MWU). Recommending self-help groups was significantly related to self-reported competence in the GP sample (*p *= 0.001, Kendall's τ), but not in the specialist sample. Less en vogue was suggesting "to the relatives that they contact the Alzheimer Association". This was reported by 52 percent of the GPs and 59 percent of the specialists (*p *= 0.005, MWU). These figures might reflect a lower degree of knowledge concerning the existence and role of the Alzheimer Association.

### The need for clinical guidelines and continuing education

The majority of GPs (56 percent) expressed the opinion that "guidelines for the diagnosis and treatment of dementia would be of help" and half (50 percent) stated an interest "in participating in a training program on how to deal and speak with demented patients and their relatives". In contrast, only a minority of the specialists were interested in guidelines (40 percent) or training (30 percent). These differences between GPs and specialists were highly significant (*p *< 0.001, MWU). GPs with lower self-perceived competence tended to be more interested in guidelines and continuing education (*p *= 0.010 and 0.011 respectively; Kendalls' τ). A greater interest in guidelines was also expressed by specialists with lower self-perceived competence (*p *= 0.012, Kendall's τ). As a whole, these results show a trend of an interrelationship between subjective competence level and interest in professional training.

### Disentangling the determinants of professional approach

In order to disentangle the various factors which influence the professional approach of physicians to dementia, an ordinal regression analysis was performed with the professional approach items as target variables (Items 5–24 in Tables 1 and 3), and self-estimated competence, attitude cluster, sex, discipline, number of patients treated and working years in ambulatory care as influencing variables. Considering p values ≤ 0.005 as significant, self-estimated competence and attitude cluster had the greatest influence on professional approach:

• Physicians with a positive attitude towards dementia care were more in favour of early detection, expressed more supportive behaviour towards relatives and felt that patients were grateful when their cognitive decline was addressed (p < 0.001 for the influence of attitudes on Items 5, 6, 11–13 and 17).

• Physicians with a higher level of self-reported competence more actively searched for dementia, were more in favour of diagnosis disclosure and expressed less interest in training and guidelines (p < 0.005 for the influence of competence on Items 7, 8, 14, 19, 20).

The remaining variables had less influence on professional approach:

• Physicians with a higher number of working years in ambulatory care expressed more reservation towards diagnosis disclosure (p ≤ 0.001 for Items 16 and 19). This was also true of female physicians (p = 0.003 for Item 21).

• The only significant contribution of the variable 'medical discipline' was that specialists more often reported employing cognitive tests (p = 0.001 for Item 8).

• The number of dementia patients treated had no significant influence on the professional approach items.

In summary, self-estimated competence and general attitude towards dementia care appear to have a considerable impact on the professional approach of physicians to the care of dementia patients. This supports the hypothetical central position of these variables shown in Figure 1.

## Discussion

Both GPs and specialists in statutory ambulatory health care in Germany felt qualified to diagnose and treat dementia. As expected, a statistically significant difference was found between specialists and GPs with respect to self-estimated competence, with specialists reporting a higher level of competence. Major differences between GPs and specialists were not found with regard to general attitude towards the care of patients with dementia: in both professions, approximately 40 percent reported a highly positive attitude, approximately 15 percent a highly negative and approximately 45 percent an ambivalent attitude. Comparable results have been reported for British GPs [2] with regard to self-perceived diagnostic competence and general attitude. A positive general attitude towards dementia care has also been demonstrated for GPs in Sweden [3].

For both GPs and specialists, a statistically significant association was found between self-estimated competence and general attitude towards caring for patients with dementia: physicians with a higher level of self-perceived competence also had a more positive attitude towards dementia care. This confirms the findings of Turner et al., who also found that GPs with a higher level of self-perceived knowledge were more optimistic with respect to dementia care [2].

In order to fully understand the differences between GPs and specialists in this study, a number of aspects peculiar to the statutory health service in Germany should be acknowledged. The health service is of a social insurance type ("Bismarkian") on the part of the client combined with a controlled market system on the part of the provider [16]. Physicians in ambulatory care work as entrepreneurs in a market characterized by unlimited direct access of patients to all disciplines without any form of gate-keeping. Task descriptions for the different medical disciplines do not exist. In general, GPs compete both among one another and with specialists. Referrals to specialists thus always have an economic in addition to a medical component, in so far as they potentially result in the loss of a patient for the GP. The degree of competition varies greatly, however, according to the degree of overlap between a specialist discipline and general practice (e.g. greater competition between GPs and paediatricians than GPs and ophthalmologists), the density of providers (e.g. more competition in large towns), and patient characteristics such as age and social status (e.g. less competition with regard to older and lower-class patients). With respect to dementia patients, competition is relatively low. It can be assumed that the majority of elderly people suffering from a developing dementia are cared for by their GP. Utilization of neurologists or psychiatrists without a GP referral is likely to be the exception, although hard data are not available. Incentives for specialists who take over the long-term care of demented patients are also lacking. Diagnosis confirmation constitutes the central reason for a GP referral to a specialist. The percentage of patients who are referred to a specialist on one or two occasions for diagnostic purposes is unknown, although it is likely to be lower than 50 percent. Memory clinics and other specialized psychogeriatric services in ambulatory care are rare; here too, the percentage of patient coverage is unknown though certainly low.

The above described health care system appears to largely explain the observed differences in approach between the two professional groups, regardless of comparable competence levels or similar general attitudes. For GPs, a higher competence level and a more positive general attitude were associated with a positive approach to single tasks (e.g. favouring early detection, active case finding and cognitive testing), a more positive attitude towards self-help services and a greater willingness to communicate and cooperate with caregivers. For specialists, associations with general attitude were found for favouring early detection, active case finding and cognitive testing but not for the items pertaining to self-help or the support of caregivers. Furthermore, no relationship was found between professional approach and self-estimated competence for specialists, with the exception of the use of cognitive tests in the case of suspected dementia. We assume that these differences between GPs and specialists correspond to their specific roles within the German care chain, in which the specialist is largely responsible for the diagnosis, whereas the provision of practical help for caregivers in providing daily care and the management of the care process remain the task of the GP.

System-based task differences may also be responsible for differences between GPs and specialists with regard to the practice of diagnosis disclosure. For the most part, specialists are seen by patients upon referral at the very end of a long process of guessing and doubting on the part of the patient, the relatives and the GP. The purpose of this referral is the establishment of a definite diagnosis. In such a situation, the use of specific terms is appropriate. This is less the case in the pre-diagnostic phase largely carried out by the GP, in which the use of vague and circumscribing terms may be equally adequate. An association between general attitude towards dementia care and handling of the disclosure process was found for neither GPs nor specialists. This finding suggests that the use or non-use of terms such as "Alzheimer" or "dementia" in the process of disclosure is not only a matter of the specific function of a discipline within the care process, but is also a domain burdened by elements of taboo. This taboo may be difficult to handle even for competent GPs who have a positive attitude towards dementia care and are basically in favour of disclosure. The tendency to inform the relatives more than the patient irrespective of self-estimated competence and general attitude in both professions also supports this hypothesis.

In general, the minor differences observed between GPs and specialists with regard to diagnosis disclosure confirm the results of De Lepeleire et al. [7]. The lack of an association between self-estimated competence and general attitude on the one hand and reported disclosure practice on the other hand found in the GP population in the present study contrasts with the findings of other studies [2,17].

Given their specific interest in guidelines and more training opportunities, it interestingly appears to be the case that those physicians who reported a lower level of competence in the present study are aware of their problem. In accordance with other authors [14,17,18], the results of the present study can be summarised as a comprehensive plea for an intensification of physician training in the area of dementia. In the study by Turner et al., GPs reported that the communication of the dementia diagnosis represented their most difficult problem [2]. The present study shows that this apparently continues to be the case even in the presence of professional competence and a positive general attitude towards caring for such patients. Training programs should therefore not only discuss the medical aspects of the diagnosis of 'dementia', but in particular topics such as overcoming related stigmatisation and taboo as well as the breaking of bad news [19-21]. Emphasis should also be placed on enhancing the general attitude towards dementia care. This suggestion is based on the results of an ordinal regression analysis showing that both self-estimated competence and attitude have more impact on professional approach than professional experience and gender.

Several limitations of the present study should be pointed out. While the samples are relatively large, they are probably not representative of the respective professional populations. Participants stem from metropolitan areas, so that the perspective of physicians in rural and remote locations is excluded. The samples may include more dedicated and interested members of both professional groups. A survey clearly has limits in terms of capturing actual behaviour. Social desirability can lead to an over-reporting of positive attitudes in response to questions related to professional standards and behaviour. Furthermore, self-estimated competence is not identical with externally observed or measured competence. Finally, the less pronounced association between competence and professional approach within the specialist sample may be due to the lower degree of variance in self-estimated competence observed in this group (see Figure 2).

The comparison of competence carried out between GPs and specialists in the present paper was not performed in order to establish which group is the best one. The focus was rather placed on the various differences within and between the two groups of physicians involved in the ambulatory care of patients with dementia. In our study, GPs were found to show a greater variance of competence than the specialists, the latter of which felt that they were more qualified and also formed a more homogeneous group. A comparative value judgement on qualification should, however, not be drawn on the basis of these results. GPs are responsible for caring for a broad spectrum of chronic diseases and patient groups, including providing medical, psychological, social and managerial support to their patients. In contrast, specialists are more able to make a definite diagnosis and treat complicated cases.

## Conclusion

A positive association was found between self-estimated competence and general attitude towards caring for patients with dementia in both GPs and specialists. For GPs, a higher level of self-estimated competence and a positive general attitude were both associated with an active approach to different tasks within the care process. Therefore, training opportunities which enable in particular GPs to enhance not only their competence but also their general attitude would appear to be beneficial and might carry positive consequences for patients with dementia and their caregivers. Differences between GPs and specialists were smaller than expected and mainly appear to reflect task differences within the German health care system.

## Competing interests

The author(s) declare that they have no competing interests.

## Authors' contributions

HK and HvdB jointly designed the study and developed the questionnaire. HK organised data collection. HK wrote an analysis plan and BW performed statistical analyses of the data. HvdB wrote the first draft of the manuscript. HK revised the first draft and worded the final version. HK was the principal author of the paper, had full access to all data, and is guarantor.

## Pre-publication history

The pre-publication history for this paper can be accessed here:


